# Functional insights of an uncommon hypomorphic variant in *IL2RG* as a monogenic cause of CVID-like disease with antibody deficiency and T CD4 lymphopenia

**DOI:** 10.3389/fimmu.2025.1544863

**Published:** 2025-03-18

**Authors:** Andrea González-Torbay, Keren Reche-Yebra, Álvaro Clemente-Bernal, Yolanda Soto Serrano, Luz Yadira Bravo-Gallego, Almudena Fernández López, Rebeca Rodríguez-Pena, María Bravo García-Morato, Eduardo López-Granados, Lucía del Pino-Molina

**Affiliations:** ^1^ Center for Biomedical Network Research on Rare Diseases (CIBERER U767), Madrid, Spain; ^2^ Lymphocyte Pathophysiology in Immunodeficiencies Group, La Paz Institute for Health Research(IdiPAZ), Madrid, Spain; ^3^ Clinical Immunology Department, La Paz University Hospital, Madrid, Spain; ^4^ Research on Comprehensive Care for Transplanted Children and Adolescent Group, La Paz Institute for Health Reserach (IdiPAZ), Madrid, Spain; ^5^ Center for Biomedical Network Research on Rare Diseases (CIBERER U756), Madrid, Spain; ^6^ Department of Molecular and Cellular Biology, National Centre for Biotechnology (CNB-CSIC), Madrid, Spain

**Keywords:** hypomorphic variants, IL2RG gene, common cytokine receptor γ chain, flow cytometry standardization, STAT3 and STAT5 phosphorylation, T and B cell proliferation, plasmablast differentiation, NK degranulation

## Abstract

**Background:**

Over the last decade, the identification of hypomorphic variants in patients previously diagnosed with Common Variable Immunodeficiency (CVID) has led to the association of milder phenotypes with variants of the *IL2RG* gene that are usually related to severe combined immunodeficiency. Indeed, several revertant mosaicisms have been described in cases with hypomorphic variants in that gene. Our main objective herein was the functional characterization of p. (Pro58Thr) variant in the *IL2RG* gene in an adult patient with antibody deficiency and moderate CD4^+^ T cell lymphopenia.

**Methods:**

Evaluation of the patient included a clinical examination and a complete analysis of the peripheral blood phenotype. To further explore *IL2RG* functionality we selected downstream signaling readouts, namely STAT3 and STAT5 phosphorylation, NK degranulation and B- and T-cell proliferation capacity *in vitro*, which can be measured by flow cytometry, that reflect the strength of homeostatic signaling pathways in resting cells and after activation.

**Results:**

The patient presented reduced CD132 expression and conserved T- and B-cell proliferation capacity *in vitro*. However, we found that intracellular signaling downstream of IL2γc is affected, with reduced STAT3 phosphorylation after IL-21 stimulation in B cells and CD4 T cells. In addition, CD4^+^ T cells showed a reduced STAT5 phosphorylation in response to IL-2, which was not so evident in CD8^+^ T cells. NK degranulation was impaired upon PHA and IL-2 as well as plasmablast differentiation *in vitro*.

**Conclusion:**

We conclude that p. (Pro58Thr) in the *IL2RG* gene is functionally a hypomorphic variant, as reported previously. Although the functionality of CD8^+^ is less impaired than the rest of the lymphocyte subsets, we did not detect a reversion of the variant in isolated CD8^+^, CD4^+^, CD19^+^ or NK cells.

## 1 Introduction

Common Variable Immunodeficiency (CVID) is the most prevalent symptomatic disease within the Inborn Errors of Immunity (IEI) disorders (1). Since its first definition in the 1970s as a primary antibody deficiency, the term CVID has evolved, and new diagnostic criteria have been introduced, to exclude for instance late onset combined immune deficiencies (LOCID) for those patients with significant T cell defects (2), and gradually some monogenic defects became associated with CVID. However, the major revolution came with the introduction of next generation sequencing (NGS) (3). Thus, several cohorts of CVID patients were progressively analyzed by NGS, which led to the definition of new monogenic immunodeficiencies extracted from the CVID definition, and the emergence of hypomorphic variants in genes known to cause severe combined immunodeficiency (SCID) with milder phenotypes than previously reported that fell within the clinical and immunological definition of CVID (4). In both situations, the need for pathogenic insights of new gene variants emerged, based on protein expression and functional assays, to determine the impact of these variants (5). Those assays frequently exceed the capability of routine laboratories, whereas associated translational research laboratories can contribute to this pathogenic insight by in-house functional assays. However, strict quality control procedures to ensure reproducibility and robustness, and inclusion of proper cohorts of healthy controls to account for inter- and intra-individual variation are required. Our research group has broad experience in immune cell signaling assessment by intracellular flow cytometry (IMMUNE SIGNAL^®^) (6–8), taking into account several procedural considerations (9). In this regard, we have leveraged the recent finding of an hypomorphic variant on the interleukin 2 receptor subunit γ or common γ chain (*IL2RG*) gene in a patient with antibody deficiency and CD4 lymphopenia followed in our clinic for more than three decades to explore the functional alteration by several non-routine assays.

The *IL2RG* gene is located on the X chromosome and codes for the common cytokine receptor γ chain (CD132) for a family of cytokines including interleukin (IL) 2, 4, 7, 9, 15, and 21. This cytokine family is involved in different key immune cell development processes, including growth, differentiation, and survival, through the activation of different signaling pathways such as Janus kinase (JAK), mitogen-activated protein kinase (MAPK), and the signal transducers and activators of transcription (STAT) proteins (10).

Pathogenic variants on the *IL2RG* gene are typically associated with X-linked SCID (T-B+NK- SCID) (11), an immunodeficiency that profoundly affects both cellular and humoral immunity. Nonetheless, several atypical cases have been reported since this entity was first described, including hypomorphic variants with non-classical phenotypes (12), patients with normal NK cells (13), late onset SCID (14), and patients with restored T cell function due to somatic reversions in T cells (15) or in common lymphoid progenitors (16).

Clinical evaluation of the patient included a complete analysis of the lymphocyte phenotype from peripheral blood. In this paper, to determine the pathogenicity of this variant, we designed a series of functional T- B and NK-cell assays to test the functional impact in this patient. We studied the protein expression of CD132 (*IL2RG* gene), the signaling downstream common cytokine receptor γ chain (γc), by STAT5 and STAT3 phosphorylation at baseline and after activation using flow cytometry. We also assessed T- and B-cell proliferation and established an *in vitro* model of B-cell activation to determine the impact of the variant in B-cell differentiation by analyzing plasmablast formation and NK-cell degranulation assay.

## 2 Methods

### 2.1 Clinical case description

**Figure 1:**
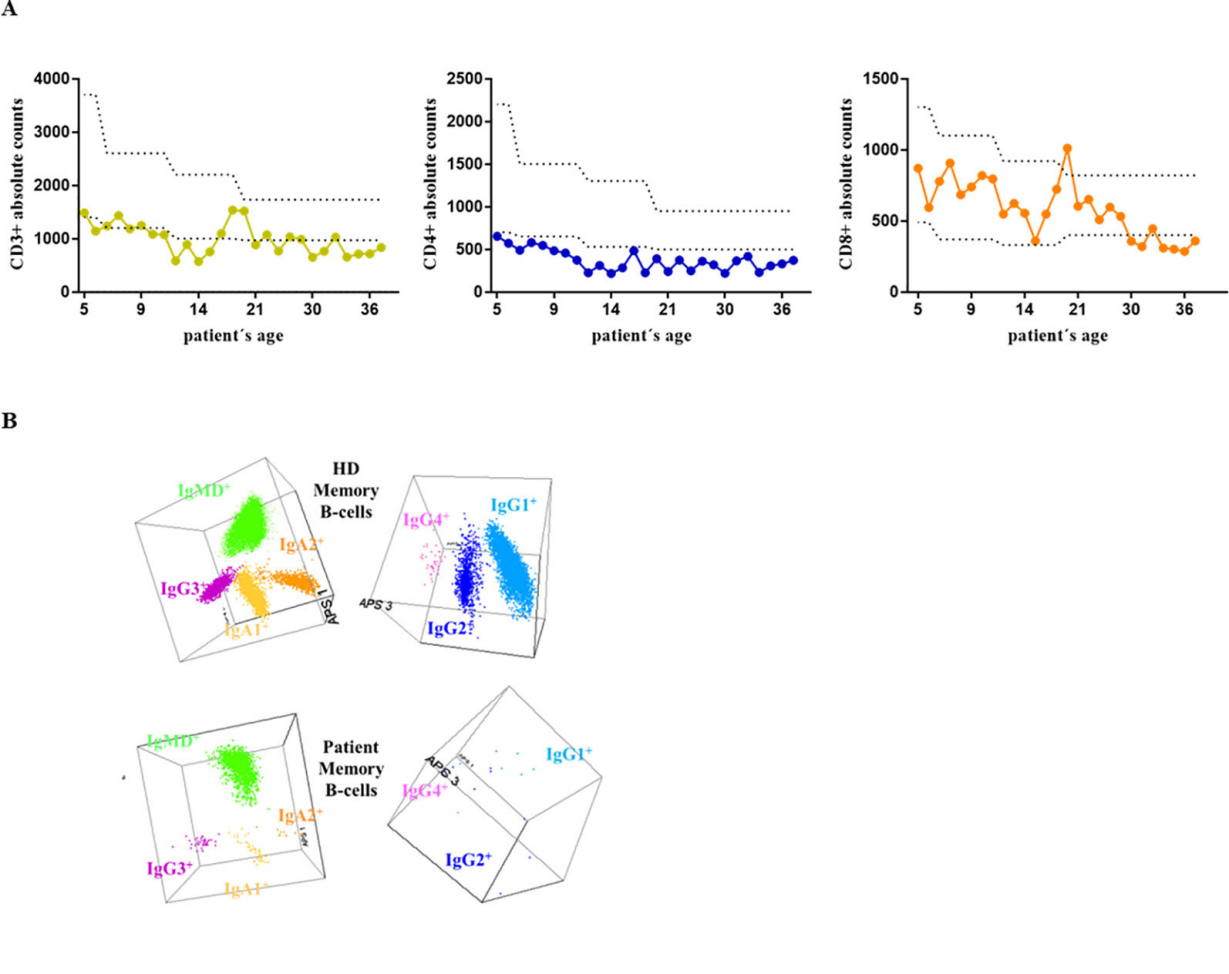
**(A)** Absolute CD3^+^, CD4^+^, and CD8^+^ counts since the patient’s first clinical record at the age of 4 until last year. **(B)** The results of the IgH isotype and subclass distribution in memory B cells (MBC) analyzed by flow cytometry in the patient and a representative healthy donor (HD). Representation of a balanced three-dimensional (3D) automated population separator (APS) diagram, constructed using the first three principal components (PC1 to PC3) derived from PC analysis (PCA) performed with the *Infinicyt* software.

A four-year-old boy was referred to our Immunology Clinic due to recurrent infections in the last 80´s. He was born on term and had grown appropriately until the age of six months, when he started experiencing loss of appetite and diarrheal episodes. At seven months, he was admitted to hospital with a *P. jirovecii* interstitial pneumonia, hypogammaglobulinemia, T-cell lymphopenia, and reduced lymphocyte proliferation in response to phytohaemagglutinin (PHA). At this moment, he was started on immunoglobulin replacement therapy (IRT) and cotrimoxazole with a presumed diagnosis of severe combined immunodeficiency. Adenosine deaminase (ADA) deficiency and purine nucleoside phosphorylase (PNP) deficiency were ruled out based on normal ADA and PNP activity. Genetic testing was not performed due to lack of availability. The boy had a history of abundant nasal discharge since birth and frequent fever episodes associated with tonsillitis. On his first visit to our clinic, lymphocyte phenotyping showed lymphocyte numbers on the lower age limit, with reduced CD4^+^ (**Table 1**, **Figure 1A**). The lymphocyte proliferation in response to PHA, concanavalin A (Con A), pokeweed mitogen (PWM) and OKT3 was normal. IRT was briefly interrupted to evaluate the production of anti-tetanus antibodies, which was absent even upon revaccination, IRT was re-started at the age of 5 years until the present time. Throughout his childhood and adolescence, he presented with several upper and lower respiratory tract infections, with evidence of bronchiectasis on chest X-ray. Adenoidectomy was performed at age six. He also had repeated episodes of conjunctivitis and dacryocystitis, which required surgical intervention. He was diagnosed with atopic dermatitis at age 14 years, and at age 18 he was admitted to the hospital with a severe *Salmonella* infection. In his twenties, he progressively had more problems with his skin, as he presented with persistent, itchy, flat warts on neck, trunk and upper limbs, diagnosed as mastocytosis, as well as rosacea, pityriasis versicolor and *molluscum contagiousum*. He remained with a presumed diagnosis of CVID-like disease with antibody deficiency and CD4 lymphopenia, until two years ago when, after informed consent was provided, genetic analysis was performed using a customized next-generation sequencing (NGS) panel with close to 400 IEI-related genes (17). This revealed a hemizygous c.172C>A, p.(Pro58Thr) variant on the interleukin 2 receptor subunit γ or common γ chain (*IL2RG*) gene, which was confirmed by Sanger sequencing and classified as probably pathogenic. In this case, the patient has an antibody deficiency phenotype compatible with CVID, although the T-cell lymphopenia, especially of naïve CD4^+^ cells and recent thymic emigrants (**Table 1**), could indicate a leaky combined immunodeficiency. The p.(Pro58Thr) variant in the *IL2RG* gene has been reported previously in a patient with hypogammaglobulinemia and diminished T cell proliferation (14), as well as another variant in the same amino-acid residue, p. (Pro58Ser), also presenting with atypical X-linked SCID (18).

**Table 1:** Summary of distribution of distinct lymphocyte populations (expressed as absolute counts or percentages) and immunoglobulin values, with reference normal values (20, 41).

	1990 (4 years old)	2002 (16 years old)	2023 (37 years old)
Lymphocytes, absolute/µL	3626 (2300-5400)	1890 (1400-3300)	**1330** (1400-3500)
CD3+, absolute/µL	1486 (1400-3700)	**756** (1000-2220)	**835** (970-1730)
CD4+, absolute/µL	**652** (700-2200)	**284** (530-1300)	**372** (500-950)
CD4+ CD45Ra+ CD31+ % CD4+ CD45 Ra+ % CD4+ CD45 Ro+ %	n.a.	n.a.	**5.07** **6.1** **76**
CD8+, absolute/µL	870 (490-1300)	359 (330-920)	**359** (400-820)
CD8+ CD45 Ra+ % CD8+ CD45 Ro+ %	n.a.	n.a.	12.3 33.3
CD3+ γδ %	n.a.	n.a.	8.41
CD16/56+, absolute/µL	n.a.	**945** (70-480)	146 (90-350)
CD19+, absolute/µL	725 (157-725)	189 (174-630)	332 (41-470)
Naïve % Unswitched memory % Switched memory % Transitionals % Plasmablasts % CD21low %	n.a.	n.a.	79,64 15,78 2,92 0,86 0,09 3,77
IgG, mg/dL	662 (480-900)	**573** (620-1150)	910 (620-1150)
IgA, mg/dL	**50** (410-1410)	59 (50-170)	58 (50-170)
IgM, mg/dL	**68** (540-1530)	**42** (55-280)	57 (55-280)
IgE, kU/L	22 (2-307)	50 (2-537)	n.a

Altered cell numbers are highlighted in bold, n.a. = data not available.

### 2.2 Blood samples from the patient and healthy control donors

Blood samples from the patient were collected during routine follow-up visits for gammaglobulin treatment at La Paz University Hospital after obtaining informed consent. Samples from healthy control donors (HD) were collected from the Blood Donation Unit of the Hospital. The study was approved by the hospital’s ethics committee (PI-2833) and adhered to the principles of the Declaration of Helsinki. Clinical data were obtained from the clinical records updated during routine medical visits for diagnosis and follow-up at the outpatient clinic of the Clinical Immunology Department.

### 2.3 Lymphocyte phenotypic analysis by flow cytometry

The distribution of distinct lymphocytes populations was assessed routinely by flow cytometry (**Table 1**, **Figure 1A**) We also explored in detail the peripheral blood B-cell compartment through specifically designed *Pre Germinal Center-B cell* and *IgH subclasses-B cell* tubes, as described previously (19, 20).

### 2.4 Flow cytometry-based analysis of T-cell proliferation

Peripheral blood mononuclear cells (PBMCs) were obtained after Ficoll density gradient centrifugation (Ficoll-Paque Premium; VWR International, Eurolab). A total of 5x10^5^ PBMCs were resuspended in prewarmed PBS+5% FCS. Cells were labeled with 2 µM Cell Trace Violet (CTV) (Invitrogen) for 15 min at 37°C, then incubated for 5 min at 37°C with 5 mL of RPMI-1640 supplemented medium (10% FCS, 100 U/mL penicillium, 100 µg/mL streptomycin and 2 mM glutamine) and washed. A total of 2.5x x10^5^ CTV^+^ cells were stimulated with 1 µg/mL of anti CD3 and anti CD28 (BD Biosciences), 2 µL of Dynabeads (Gibco) or anti CD3/CD28/CD2 activator (Stemcell) in 96-well plates and maintained for 3 and 6 days at 37°C in a 5% CO_2_ atmosphere. Unstimulated cells were processed in parallel. Cell proliferation assay was performed by flow cytometry. Cells were stained with CD3 APC, CD8 PE, CD27 FITC, CD45 RA BV510 (BD Biosciences), CD19 PeCy7 (Beckman Coulter), and Sytox AADvanced dead cell stain (Molecular probes) using Dx Flex (Beckman Coulter) and analyzed with the Flow Jo software (Flow-Jo LLC). Cell proliferation was measured as the proliferating index in different populations in comparison with unstimulated samples.

### 2.5 Flow cytometry analysis of surface CD132 expression

Whole blood was stained with CD45 KrO, CD3 ECD, CD19 PE Cy7, CD4 APC750, CD16 APC and CD56 APC (Beckman Coulter), CD8 FITC (Cytognos), CD132 PE and mouse IgG1, κ PE isotype control (BD Biosciences) for 30 min, then fixed and lysed, washed and acquired in Dx Flex (Beckman Coulter), and analyzed using Flow Jo software (Flow-Jo LLC).

### 2.6 Flow cytometry analysis of STAT5 and STAT3 phosphorylation

To test the strength of the mutated common-gamma chain intracellular signaling we measured the intracellular content of phospho-STAT5 (pSTAT5) at Y694 and phospho-STAT3 (pSTAT3) at Y705 under basal conditions and after stimulation, as per the manufacturer’s instructions. A total of 5x10^5^ PBMCs were resuspended in 250 µL RPMI-1640 supplemented medium (10% FCS, 100 U/mL penicillium, 100 µg/mL streptomycin, and 2 mM glutamine). PBMCs were left to rest at 37 °C for 30 min. The cells were then stimulated with different concentrations (10–100 U) of IL-2 (Peprotech) or with 10 ng/mL of IL-21 (Invitrogen) at 37 °C in the water bath; unstimulated samples were processed in parallel. pSTAT5 and pSTAT3 were analyzed in different tubes. Cells were then fixed with prewarmed Lyse/Fix (Phosflow, BD Biosciences) for 10 min at 37°C and permeabilized with Perm III Buffer (Phosflow, BD Biosciences) on ice according to the manufacturer’s instructions. Cells were stained with CD3 APC, CD8 PE, CD27 BV421, pSTAT5 Alexa Fluor 488 with the isotype control Mouse IgG1, κ Alexa Fluor 488 and pSTAT3 Alexa Fluor 488 with the isotype control Mouse IgG2a AF488 (BD Biosciences), CD4 APC-A750, CD19 PeCy7 (Beckman Coulter), and IgD PE (Southern Biotech). Responsiveness to IL-2 or IL-21 stimulation was calculated as the ratio of the MFI of pSTAT5 or pSTAT3, respectively, in stimulated cells to the MFI of pSTAT5 or pSTAT3 in unstimulated cells. Samples were acquired in Dx Flex (Beckman Coulter) and analyzed using the Flow Jo software (Flow-Jo LLC).

### 2.7 *In vitro* B-cell activation

Total B-cells were obtained from freshly obtained PBMCs by magnetic isolation using the B Cell Isolation Kit II (Miltenyi Biotech). B-cell enrichment was assessed using CD19 Pe Cy7 (Beckman Coulter), CD27 BV421, CD3 APC (BD Biosciences), and IgD PE (Southern Biotech). Part of the isolated B cells from the patient and HDs were labeled with 2 µM of CTV, as described above, to measure B-cell proliferation, then activated under different conditions. The remaining B cells were used to analyze plasmablast formation. Thus, 1x10^5^ purified B-cells were suspended in 200 µL supplemented RPMI-1640 medium (10% fetal calf serum, 100U/mL penicillium, 100 µg/mL streptomycin, and 2 mM glutamine). Isolated B cells with and without CTV were activated for 6 days under different stimuli combinations, for T cell dependent activation: 1 μg/mL MegaCD40L soluble human recombinant (Enzo Life Sciences) and 100 ng/mL human IL-21 recombinant protein (Gibco, Life Technologies), and a mixture of T cell dependent and independent combination stimuli adapted from Le Gallou et al (21), consisting of 3 ng/mL recombinant human IL-2 (Peprotech), 2.5 µg/mL CpG oligonucleotide (ODN 2006 TLR9 ligand, Invivogen), 2 µg/mL AffiniPure F(ab´)_2_ fragment Goat anti Human IgA+IgG+IgM (H+L) (Jackson ImmunoResearch) and 1 μg/mL MegaCD40L, then restimulated at day 3 with IL-21 (100 ng/mL), IL-4 (10 ng/mL), and IL2 (6 ng/mL). Unstimulated samples were performed in parallel. Cell cultures were maintained for 6 days at 37°C in a 5% CO_2_ atmosphere. For the analysis of B cell phenotype, B cells were stained with CD27 FITC, IgG PE, CD38 APC-H7 (BD Biosciences), CD19 PeCy7 (Beckman Coulter), IgD A700 and IgM BV510 (Biolegend), IgA APC (Miltenyi), and Sytox AADvanced dead cell stain (Molecular probes). The analysis was performed by flow cytometry using DxFlex (Beckman Coulter) and the Flow Jo Software (LLC). B-cell proliferation was measured as the proliferation index in stimulated conditions in comparison with unstimulated samples.

### 2.8 NK degranulation

We performed two different protocols for NK-cell degranulation assay in resting conditions and after stimulation with PHA/IL2 for 2 days, as previously described (22). 2x10^5^ PBMCs in fresh resting conditions or preactivated for 2 days with PHA/IL2 were incubated with 2x10^5^ K562 target cells at 37°C for 3 hours in 96 well-bottom plates. Cells were stained with CD56 APC, CD3 ECD, CD16 FITC, CD8 APC-A700, CD107a Pacific Blue and CD45 KO antibodies (Beckman Coulter). The analysis was performed by flow cytometry using DxFlex (Beckman Coulter) and the Flow Jo Software (LLC).

### 2.9 Sanger sequencing in DNA isolated from CD4^+^, CD8^+^, CD19^+^ and NK cells

PBMCs were stained with CD3 ECD, CD19 PE Cy7, CD4 APC750, CD16 APC and CD56 APC (Beckman Coulter), and CD8 FITC (Cytognos) antibodies, and CD4^+^, CD8^+^, CD19^+^, and NK cells were sorted using a MoFlo Astrios sorter (Beckman Coulter). DNA was extracted using the QIAamp DNA micro kit. Genomic DNA in the target region was amplified using PCR master mix DNA polymerase (Life Technologies) in a 2720 Thermal Cycle (Applied Biosystems) and with GC-rich PCR system (Roche) for exon with high content of GC. The primers (Sigma-Aldrich) were designed with SnapGene software and Primer Blast resources. The sequences of the primers used are listed in **Supplementary Table S1**. The PCR product was treated with Ex´S-Pure (Nimagen). Sanger sequencing of the gene variant in CD4^+^, CD8^+^, CD19^+^, and NK cells was performed on a non-diagnostic facility on a research basis.

### 2.10 General considerations for in-house functional assays based on flow cytometry

Our research group has broad experience in immune cell signaling assessment by intracellular flow cytometry (IMMUNE SIGNAL^®^), and we are familiar with the principles of standardization and quality control in routine diagnostic settings. A well-defined experimental procedure and appropriate cytometer set up is key for the robustness of non-routine functional assays. To that end, we performed daily quality-control checks DxFlex Daily QC Fluorospheres (Beckman Coulter) to ensure that the flow cytometer instrument was working with adequate signal strength and precision. For standardization of the DxFlex flow cytometer, we used Flow-Set Pro fluorospheres (Beckman Coulter). For each specific assay, we defined the target median values or median fluorescence intensities (MFIs), updated daily with Flow-Set Pro fluorospheres, with adjustment of the optimized gain settings to generate the target median values or MFI. In this way, we could progressively accumulate results from healthy control participants, thus leading to a good representation of biological variation among individuals, controlling the intrinsic inter-assay variability by means of the daily quality-control check and updating of the cytometer settings. Moreover, we already exemplified the validity of our approach to discriminate biological differences in the phosphorylation status of targeted proteins in primary B- and T-cells between a range of normal controls and genetically confirmed IEIs patients (23).

### 2.11 Statistics

Data analysis was performed using GraphPad Prism version 9.0 software (San Diego, CA, USA). Statistical differences between the patient and controls were determined by applying the nonparametric Mann–Whitney test. Differences were considered statistically significant for p-values of * <0.05, ** <0.01 and *** <0.001.

## 3 Results

### 3.1 Phenotypic analysis of lymphocytes, CD132 expression and T cell proliferation

Retrospective results of the routine clinical evaluation of the patient along the years, included analysis of the lymphocyte phenotype from peripheral blood, summarized in **Table 1**. The patient presents T-cell lymphopenia (**Figure 1A**), mostly related to reduced naïve CD4^+^ cells and recent thymic emigrants (RTE). Similarly, although total B-cell counts are in normal range, the memory B cell (MBC) compartment is in the lower limit of normality, with almost normal numbers of unswitched IgMD^+^ MBC, but reduced switched IgG1^+^, IgG2^+^, IgA1^+^ MBC, IgG3^+^ at the lower limit, and undetectable IgA2^+^ and IgG4^+^ MBC (**Figure 1B**). The plasma cell compartment is severely reduced, with predominating IgM^+^ plasma cells, normal absolute IgG2^+^ counts, low IgA1^+^, and the remaining isotypes being absent.

**Figure 2:**
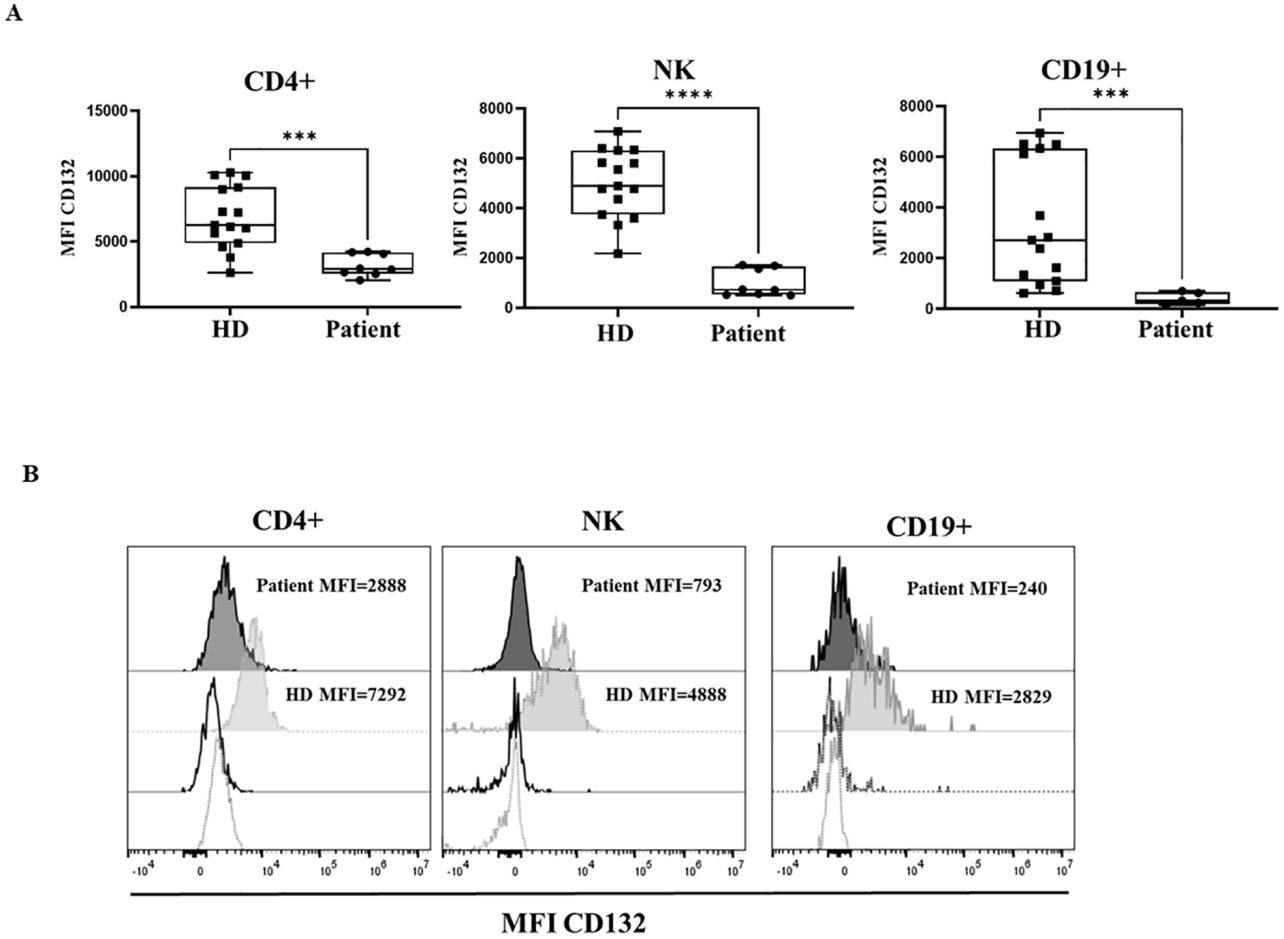
CD132 expression. **(A)** Median fluorescence intensity (MFI) of CD132 in healthy donors (HD) and the patient with the variant p. Pro58Thr in the *IL2RG* gene, in CD4^+^, NK and B cells. Patient samples were processed in triplicates in three independent experiments. **(B)** Histograms show MFI of CD132 in a representative HD (grey) and the patient (black), with dotted lines showing isotype controls. P-values <0.05 were considered to have statistical significance and are coded as follows: ***p<0.001; ****p<0.0001.

γc surface expression on T, B and NK cells was measured by flow cytometry with an anti- CD132 antibody. The patient’s CD4^+^, B cells and NK cells showed a significant reduction in CD132 surface expression when compared to healthy donors (**Figures 2A, B**). Also, CD132 surface expression was reduced in monocytes and granulocytes, selected by forward/scatter properties and CD45^+^ expression, in the patient in comparison to healthy donors (**Supplementary Figure S1**).

**Figure 3:**
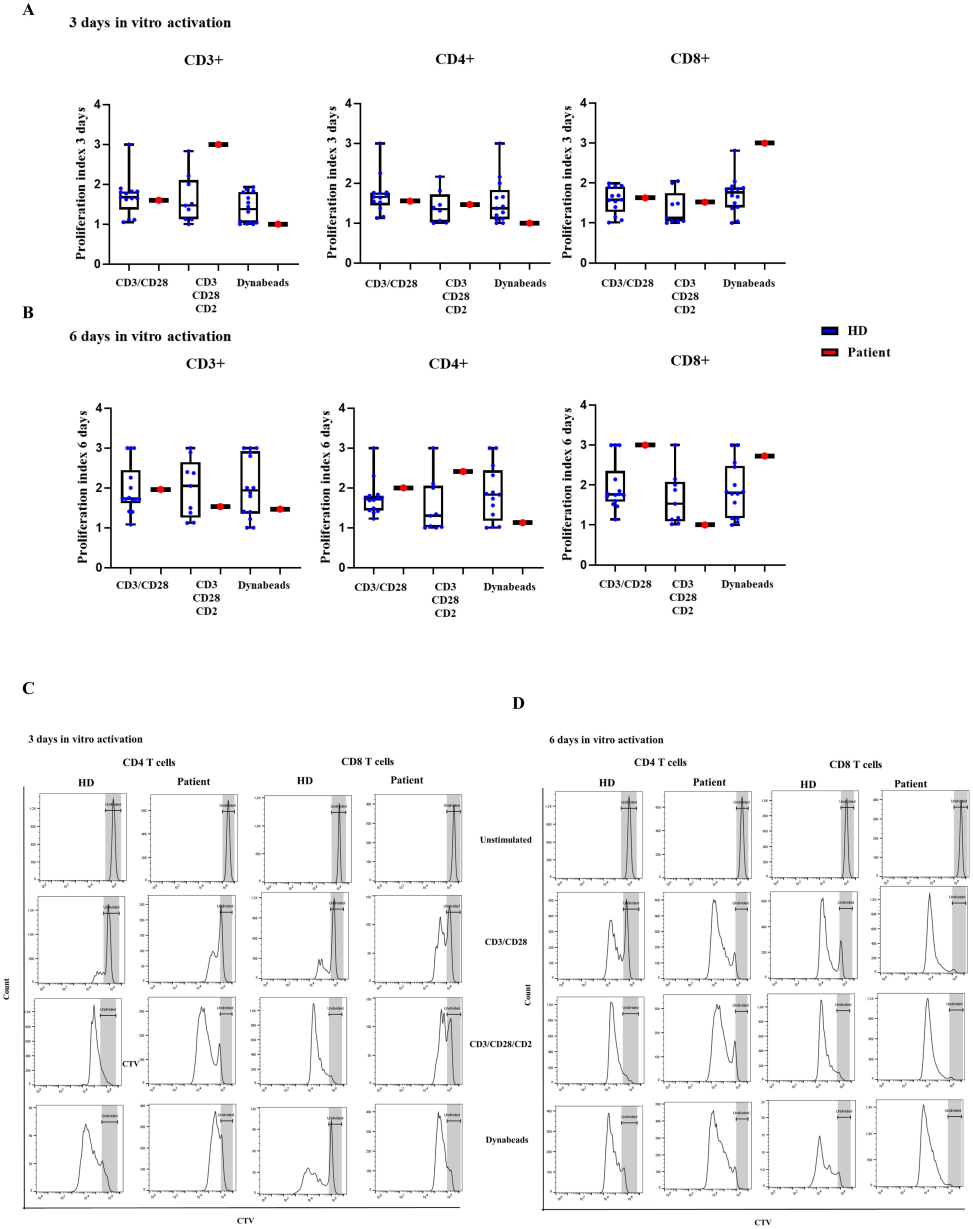
T-cell proliferation. Proliferating index measured with CTV-labelled CD3^+^, CD4^+^ and CD8^+^ T cells in healthy donors (HD) and the patient with the variant p. Pro58Thr in the *IL2RG* gene after activation for **(A)** 3 and **(B)** 6 days with CD3/CD28, CD3/CD28/CD2/and dynabeads (DN). **(C)** Histograms of representative healthy donor (HD) and patient, showing the proliferating cells at rest and after activation for 3 and **(D)** 6 days with CD3/CD28, CD3/CD28/CD2/and dynabeads (DN).

Normal routine T-cell lymphoproliferation assays, based of tritium incorporation in response to mitogens, were evidenced for several years during patient follow-up. We performed a more comprehensive analysis of the lymphoproliferative capacity based on the cell trace violet (CTV) dye, and three different reagents that provide TCR primary and co-stimulatory signals for T cell activation (CD3/CD28 as monoclonal antibody or dynabeads, and CD3/CD28/CD2 T cell activator). For all three T-cell activation cocktails, CD4^+^ and CD8^+^ T-cells from the patient showed normal proliferation at day 3 of stimulation (**Figures 3A, C**) and at day 6 of stimulation in comparison to HD (**Figures 3B, D**).

### 3.2 Intracellular analysis of STAT3 and STAT5 phosphorylation

**Figure 4:**
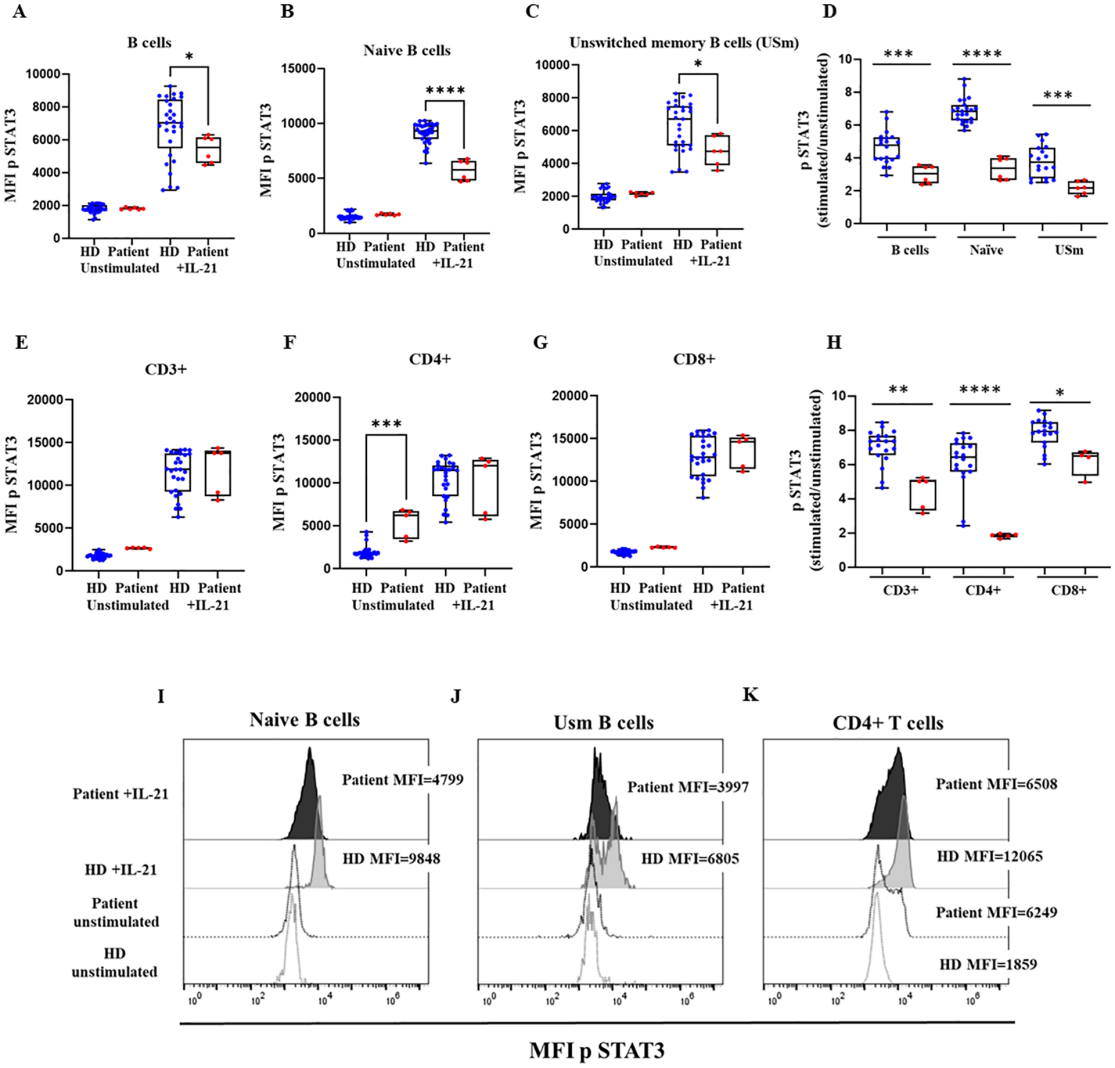
STAT3 phosphorylation. Median fluorescence intensity (MFI) of STAT3 phosphorylation in healthy donors (HD) and the patient with the variant p. Pro58Thr in the *IL2RG* gene in CD19^+^ **(A)**, naïve B-cells **(B)** and unswitched memory B cells (USm) **(C)**, and the ratio of STAT3 phosphorylation after IL-21 activation **(D)**. MFI of STAT3 phosphorylation in CD3^+^ **(E)**, CD4^+^ **(F)**, and CD8^+^ T cells **(G)**, and the ratio of STAT3 phosphorylation after IL-21 activation **(H)**. The patient’s samples are processed in triplicate in three independent experiments. Histograms of representative HD (in grey) and patient (in black) showing MFI of STAT3 phosphorylation at baseline (dotted lines) and after activation with IL-21 in naïve B cells **(I)**, USm B cells **(J)** and CD4^+^ T cells **(K)**. P-values <0.05 were considered to have statistical significance and are coded as follows: *p<0.05; **p<0.01; ***p<0.001; ****p<0.0001.

The intracellular signaling downstream the common cytokine receptor γ chain (γc) was studied in order to evaluate the pathogenic impact of the Pro58Thr variant in the *IL2RG* gene. To that end, STAT3 phosphorylation was analyzed by flow cytometry after IL-21 stimulation of B- and T-cells. We found severely reduced STAT3 phosphorylation in naïve B cells and unswitched memory B cells from the patient (**Figures 4A–D, I, J**). STAT3 phosphorylation was also reduced in T cells (**Figure 4E**), CD4^+^ T cells presented high basal phosphorylation levels of STAT3 and, in consequence, the induction after IL-2 stimulation was reduced in comparison to HD (**Figures 4F, H, K**). This reduced induction was not so marked in CD8^+^ T cells (**Figure 4G**).

**Figure 5:**
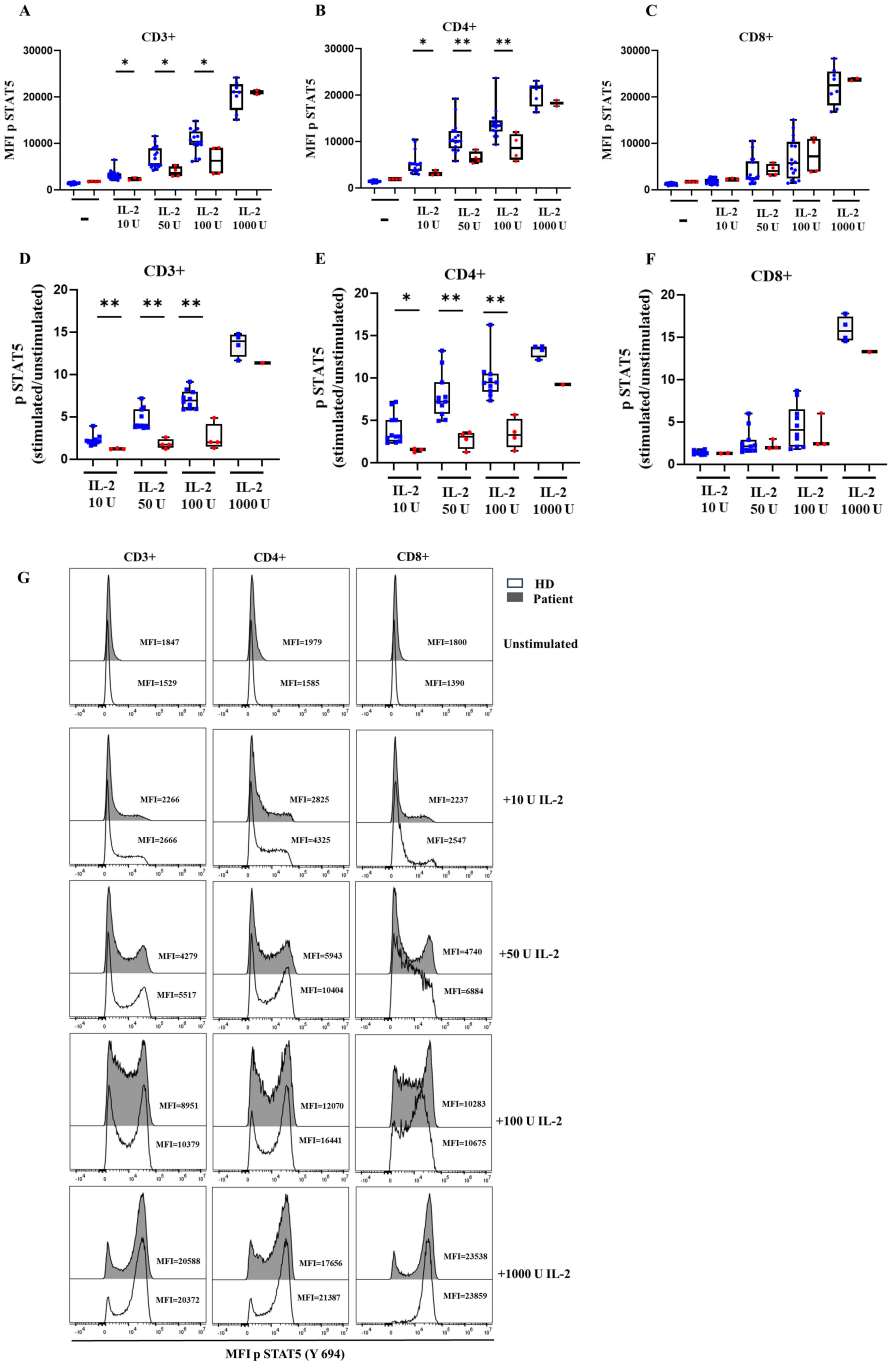
STAT5 phosphorylation. Median fluorescence intensity (MFI) of STAT5 phosphorylation and ratio at baseline and after IL-2 stimulation for healthy donors (HD) and the patient with the variant p. Pro58Thr in the *IL2RG* gene in CD3^+^ **(A)**, CD4^+^ **(B)**, and CD8^+^ T cells **(C)** and the ratio of STAT5 phosphorylation after IL-2 activation in CD3^+^ **(D)**, CD4^+^ **(E)**, and CD8^+^ **(F)** T cells. The patient’s samples are processed in triplicate in three independent experiments. **(G)** Histograms of representative HD and patient showing the MFI for STAT5 phosphorylation at baseline and after activation with IL-2 at different concentrations. P-values <0.05 were considered to have statistical significance and are coded as follows: *p<0.05; **p<0.01.

We also obtained other γc signaling data by a dose-response analysis of STAT5 phosphorylation in the presence of increasing amounts of IL-2 (10–100U) in total CD3+, CD4+ and CD8+ T cells (**Figures 5A–C**), and the ratio of phosphorylation (**Figures 5D–F**) with the corresponding histograms (**Figure 5G**). We found reduced MFI levels of pSTAT5 in CD4^+^ T cells with low doses of IL-2 (10, 50 and 100 U/mL) compared to HD, and normal MFI pSTAT5 levels with higher doses of IL-2 (1000 U/mL) (**Figure 5B**). However, a more focused analysis of activation-induced levels of pSTAT5 as a fold induction after increasing amounts of IL-2 reveals a reduction in comparison to HD (**Figure 5E**). Again, this phosphorylation defect is not evident in CD8^+^ T cells from the patient, which only showed reduced fold induction of STAT5 phosphorylation when compared to age-related HD at higher IL-2 concentration levels (**Figures 5C, F**).

### 3.3 *In vitro* induced B cell differentiation and proliferation

**Figure 6:**
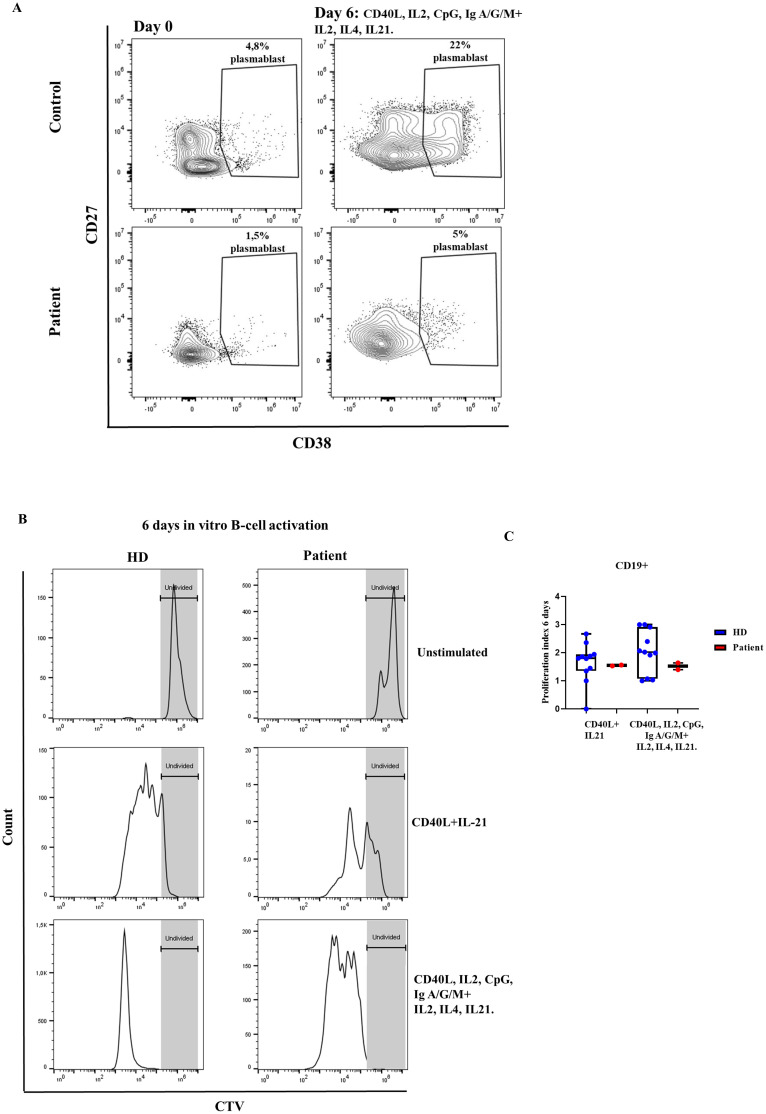
B-cell activation. **(A)** Percentage of plasmablast formation (CD38^++^ CD27^+^), with CD40L, IL2, CpG, Ig A/G/M, and restimulated at day 3 with IL21, IL4 and IL2. The patient’s samples are from three independent experiments. **(B)** Histograms of representative healthy donor (HD) and patient with the variant p. Pro58Thr in the *IL2RG* gene, showing the proliferation index **(C)** at rest and after activation for 6 days with different combinations of stimuli.

For the *in vitro* B cell activation we tested two different combinations of stimuli, namely T cell dependent stimuli (CD40L, IL-21) and of T cell dependent and independent stimuli (CD40L, IL-2, CpG, anti-IgG/IgA/IgM F (ab′)_2_ and restimulation at day 3 with IL-21, IL-4 and IL2). With the latter, we established day +6 of *in vitro* B cell activation as the one in which cells from HD reached a higher percentage of plasmablasts (CD38^++^ CD27^+^) (data not shown). However, with this combination of T cell dependent and independent stimuli, the patient failed to differentiate to plasmablasts (CD38^++^ CD27^+^) (**Figure 6A**). Whereas, B cell proliferation, as measured by the proliferation index, was conserved in the patient after stimulation with both T cell dependent stimuli and the combination of T cell dependent and independent stimuli (**Figures 6B, C**).

### 3.4 NK-cell degranulation assay

**Figure 7:**
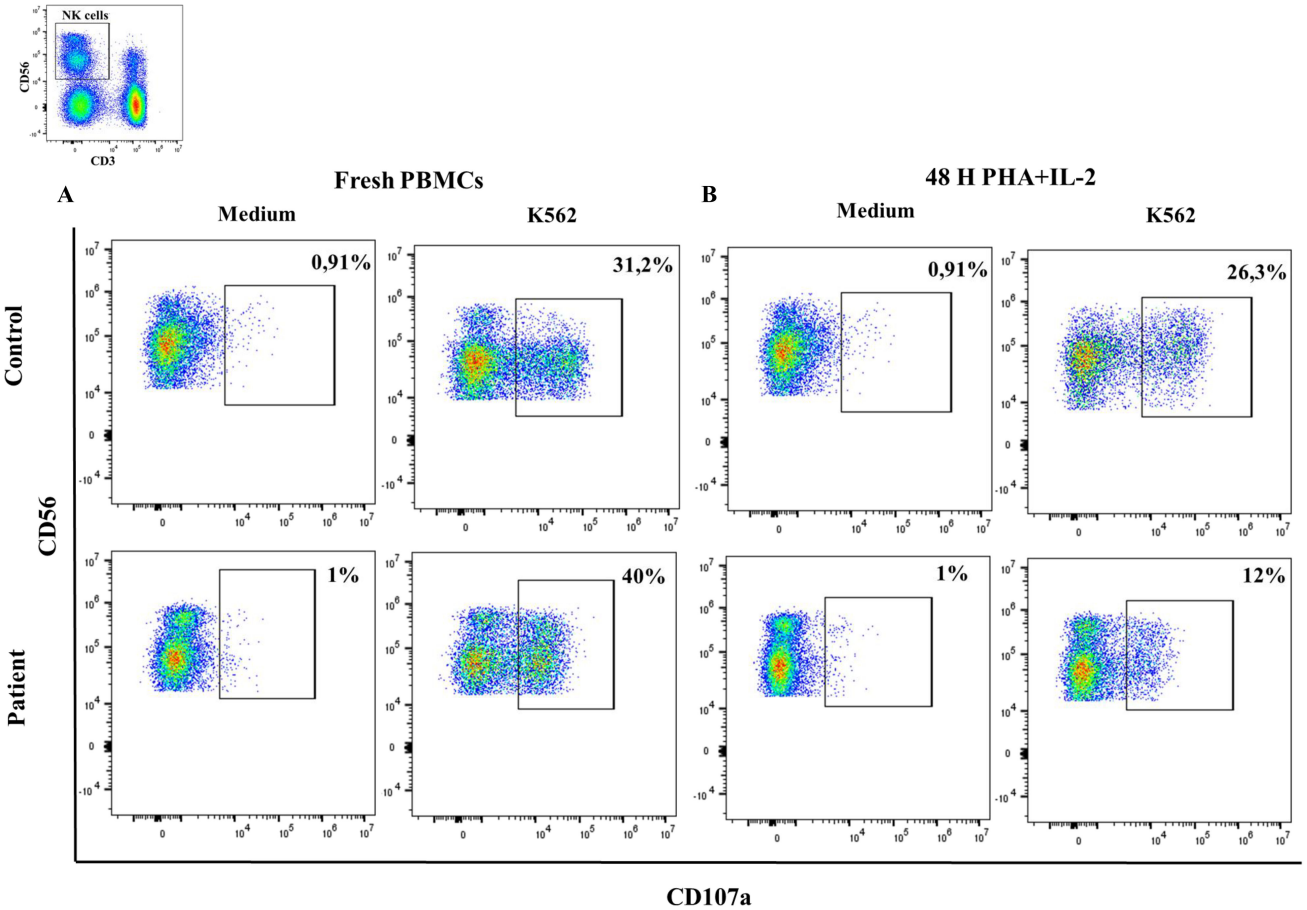
NK-cell degranulation. Percentage of CD107a expression in CD3^-^CD56^+^ cells in resting fresh PBMCs **(A)** or stimulated with PHA/IL2 for 2 days **(B)** at baseline and after incubation with K562 for 3 hours in a healthy donor and the patient with the variant p. Pro58Thr in the *IL2RG* gene.

We detected normal NK-cell degranulation analyzed by CD107a expression after stimulation with K562 targets in the patient (**Figure 7A**). However, after two days of NK-cell pre-stimulation with PHA/IL2, the patient presented reduced NK-cell degranulation in comparison to the healthy control (**Figure 7B**). These results indicate impaired functional responses of NK-cell of upon IL2 stimulation of the Pro58Thr variant.

### 3.5 Analysis of the variant in CD4^+^, CD8^+^, CD19^+^ and NK cells

**Figure 8:**
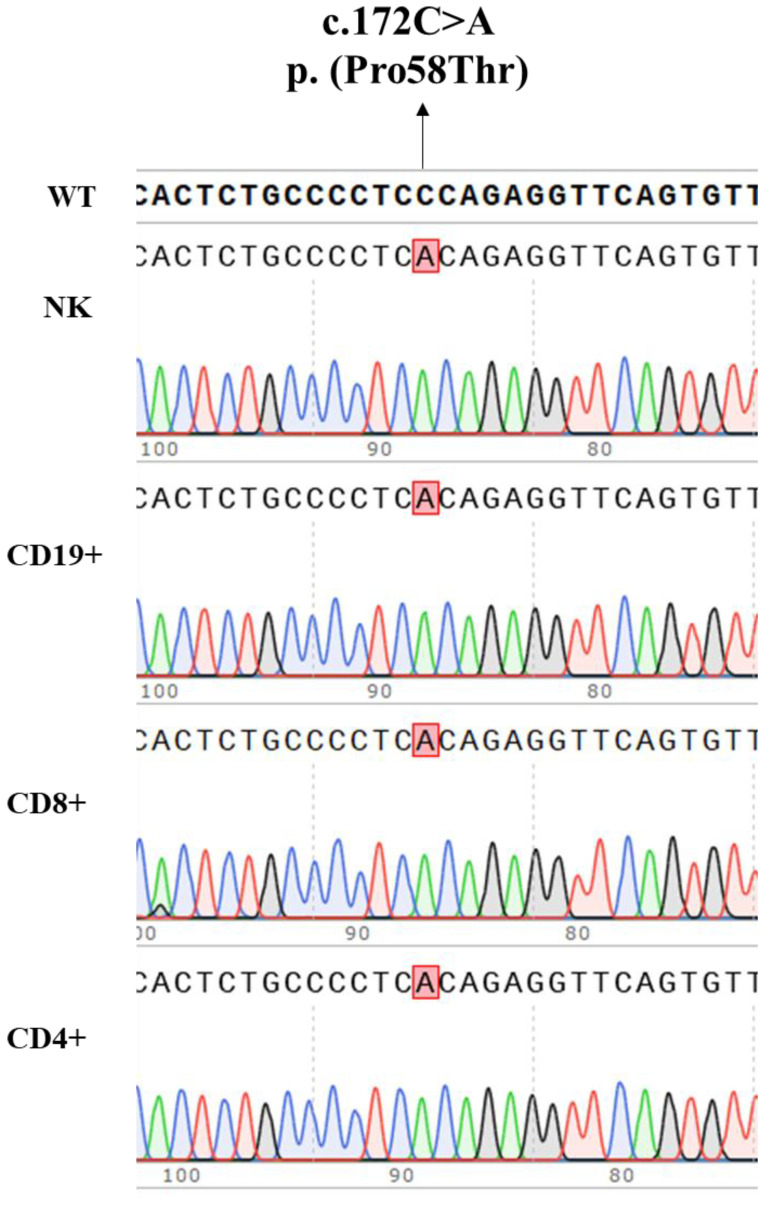
Nucleotide substitution c.172C>A, p. (Pro58Thr), in the *IL2RG* gene present in genomic DNA isolated from the patient’s CD4^+^, CD8^+^, CD19^+^ and NK cells.

To further explore a potential gene variant reversion mechanism as contributor to the milder immunological and clinical phenotype, as described in several patients with variants in *IL2RG* gene and atypical clinical presentation, we performed Sanger sequencing in sorted CD4^+^, CD8^+^, CD19^+^, and NK cells. The nucleotide substitution c.172C>A was the only present in all the lymphocyte subsets studied (**Figure 8**). Moreover, the analysis of the entire coding sequence of *IL2RG* gene did not reveal any other variant in sorted CD4^+^ and CD8^+^ T cells.

## 4 Discussion

Herein we explore the functional alteration of the hemizygous variant c.172C>A, p.(Pro58Thr) in *IL2RG* found in an adult patient who has been followed-up at our Clinical Immunology department for more than three decades for a CVID-like antibody deficiency with low T CD4^+^ counts. We performed a comprehensive analysis on the impact of this variant on protein expression and function in different lymphocyte subtypes, and its consequences for induced lymphoproliferative and differentiation capacity on cells.

The p. (Pro58Thr) related CD132 expression is reduced in CD4^+^ T cells, B and NK cells. The amino acid substitution is located at the extracellular domain of the protein. This is consistent with the reduced plasma membrane expression observed in another variant within the same amino acid residue but with different significance, p.(Pro58Ser). While *IL2RG* mRNA levels were comparable to healthy controls (18) the analysis of protein-protein interactions of mutant p.(Pro58Ser), revealed higher frequency of interactions with other proteins from the endoplasmic reticulum/Golgi apparatus and nuclear proteins, that might be altering the plasma membrane targeting, affecting IL2RG cell surface expression (18).

Although our patient presents CD4^+^ T cell lymphopenia and antibody deficiency, we assessed normal CD4^+^ and CD8^+^ cell proliferation with CD3/CD28 and in B cells with both T cell dependent stimuli (CD40L and IL-21) and the combination of T cell dependent and independent stimuli. The apparent slightly different findings regarding T cell proliferation in our patient with p.Pro58Thr variant and the patient reported by Tuovinen et al. p.Pro58Ser, could be due because we performed proliferation analysis with specific T cell stimulants (CD3/CD28), while they used directly IL-2 (18). Normal T- and B-cell proliferation has been reported in a patient with the *IL2RG* variant p.(Asp134Val), who also suffers from CD4^+^ lymphopenia and antibody deficiency (24). Although we tested T- cell proliferation supplemented with increasing doses of IL-2 *in vitro*, no difference was observed since T-cell proliferation is conserved in our patient.

IL2RG is the common cytokine receptor γ chain for several cytokines, mainly produced by different subsets of T cells, and which are involved in different immune functions given their association with JAK and STAT proteins. As such, we analyzed the phosphorylation of STAT3 and STAT5 by inducing them with IL-21 and IL-2, respectively. IL-21 promotes B-cell differentiation and Ig production and enhances follicular helper T cells (Tfh) differentiation, while IL-2 is a T-cell and NK cell growth factor that also enhances B-cell function by inducing plasma cell differentiation (10).

We found that p.(Pro58Thr) impairs STAT3 phosphorylation in response to IL-21 stimulation in different B-cell subsets and in CD4^+^ T cells. In CD4^+^ T cells we observed higher basal phosphorylation levels of STAT3, therefore the levels after stimulation with IL-21 were reduced in comparison to HD, whereas in CD8^+^ T cells the phosphorylation levels of STAT3 were not as markedly reduced as in CD4^+^ T cells. In the patient with the variant on the same amino acid residue [p.(Pro58Ser)], defective STAT3 phosphorylation was also observed in B cells (18). This same variant was also detected by newborn screening, in a second patient who had been healthy until the age of 3 years when the report was published (18).

In our patient we found defective STAT5 phosphorylation in CD4^+^ T cells with low doses of IL-2 (10, 50 and 100 U/mL) when compared with HD; these results are similar to those obtained in other patients with hypomorphic variants (12). Also, in the variant reported here p.Pro58Thr and p.Pro58Ser described by Tuovinen et al. both cases impaired STAT5 phosphorylation was assessed in response to 10U/mL of IL-2. In our case we only achieved normal STAT5 phosphorylation with high amounts of IL-2 (1000 U/mL), being up to 100 U/mL severely impaired, similar results from that obtained in p.Pro58Ser with 320 U/ml of IL-2 (18). Normal STAT5 phosphorylation levels are only achieved with higher doses of IL-2 (1000 U/mL), as detected in patients with the p.(Ile153Thr) hypomorphic variant (25).

The variant we report here, p. (Pro58Thr), has been previously described in an infant with recurrent infections, hypogammaglobulinemia, poor T cell proliferative response, reduced CD132 expression with impaired STAT5 phosphorylation, who died of graft failure after hematopoietic stem cell transplantation (14). Further studies in this patient confirmed the somatic reversion in a fraction of CD8^+^ and NK cells. We also looked for a potential gene reversion in isolated CD4^+^, CD8^+^, NK, and CD19^+^ cells by Sanger sequencing that could explain the less impaired function in CD8^+^ T cells from our patient. However, CD4^+^, CD8^+^, NK, and CD19^+^ cells presented only the nucleotide substitution c.172C>A, p. (Pro58Thr) in the *IL2RG* gene.

B-cell differentiation is severely impaired in our p.(Pro58Thr) patient, as seen by the reduced numbers of different subclasses of switched memory B cells and plasma cells. Physiologically, once naïve B cells encounter antigens in the secondary lymphoid organs, they are activated via the B cell receptor (BCR) and migrate to the border between the B cell follicular and T cell zones (26), where they can interact with T cells that have been activated by the same antigen (T-cell dependent response) or without the help of T cells (T-cell independent response) to generate memory B cells and plasma cells both from the germinal center (GC) origin or independent of GC (27). We performed a mimicking *in vitro* B-cell activation assay based on previously reported protocols (21, 28) that combine T-cell dependent and independent stimuli to effectively induce B-cell differentiation. Thus, we established a method for *in vitro* plasma cell differentiation with a sequential B-cell activation using BCR (anti IgM) and TLR9 (CpG ligand), both of which are critical for B-cell proliferation, together with *in vitro* T-cell cooperation (CD40L) and IL-2 and a second stimulation with several IL, all sharing the γ chain receptor. IL-2 is required for plasma cell development, especially during the first 48 hours (21, 29), whereas IL-4 promotes B cell survival and increases the expression of activation markers such as activation-induced cytidine deaminase (AICDA) to promote somatic hypermutation and class switch recombination (CSR) of memory B cells, to IgG1 and IgE (30). IL-21 promotes B cell survival, germinal center formation, CSR to IgG1, plasma cell differentiation, and Tfh formation (31, 32). In this study we analyzed plasmablast formation after 6 days of B-cell activation *in vitro*. *In vivo*, plasmablasts are short-lived effector cells generated during the early antibody response, whereas long-lived plasma cells promote humoral immunity (33). We found defective plasmablast formation with T-cell dependent stimuli (CD40L and IL-21). Moreover, the inclusion of IL-2 in the sequential B-cell activation method (CD40L, IL-2, CpG, anti-IgG/IgA/IgM F (ab′)_2_ and restimulated at day 3 with IL-21, IL-4, and IL2) was insufficient to promote *in vitro* plasmablast differentiation in the patient with the p.(Pro58Thr) variant. This is also seen *in vivo*, where IL-2 produced by T cells together with CD40L might not be sufficient for plasmablast differentiation. Moreover, IL-21 is also relevant for terminal B-cell differentiation via STAT3 signaling (29), which we have also found to be impaired in the patient’s B cells. All these findings may contribute to the antibody deficiency present in this patient with atypical X-linked SCID. B-cell alterations have been reported in patients with hypomorphic variants in the *IL2RG* gene, with impaired B-cell proliferation and plasmablast formation with CD40L and IL21, but normal proliferation and plasmablast differentiation with CpG and IgM, thus leading to the conclusion that γ chain dependent responses to IL-2 and IL-21 are impaired (12, 34).

Although the patient presented normal NK cell numbers, as previously reported in other hypomorphic variants in *IL2RG* gene (24), we observed a functional impairment of NK-cell degranulation upon IL-2 stimulation, similar findings was reported in atypical SCID with p. Arg222Cys variant (34).

With regard to other clinical symptoms, granulomatous infiltrations and autoimmune complications have been described in combined immunodeficiencies associated to RAG and particularly, IL2RG hypomorphic variants (35). We have not observed so far any of these in our patient.

Several somatic reversions have been described in atypical X-linked SCID, along with partially conserved T cell function (16). This leads to milder symptoms and incomplete penetrance because revertant cells present a selective advantage (36). Interestingly, the patient harboring a variant in the same residue (c.172C>T, p. (Pro58Ser)) who has recurrent infections, bronchiectasis, reduced CD132 expression, and impaired STAT5 phosphorylation with IL-2 and IL-21, but normal Ig levels and conserved antibody levels to pneumococcus and tetanus, presented an expansion of γδ T cells (18). Subsequently, the authors reported a second variant p.(Phe178Leu) in γδ T cells with normal CD132 expression and STAT5 phosphorylation. This second variant p.(Phe178Leu) partially rescues the first variant p.(Pro58Ser), and confers the cells a selective advantage (37). Expanded γδ T cells, with higher susceptibility to lymphoproliferation by chronic active EBV and CMV infection and autoimmune cytopenia, have been reported in other hypomorphic variants in the *IL2RG* gene (38, 39). The patient reported here with p.(Pro58Thr) presents normal numbers of γδ T cells.

This manuscript presents a broad set of assays that could be applied to explore the pathogenic consequences of gene sequence variants in *IL2RG*, and other related genes from this signaling pathway, which involves STATs, JAKs, multiple cytokines, and their receptors. Recent technological advances in genetics have expanded our ability to diagnose IEI, and detect the appearance of variants of uncertain significance, enormously (40). However, the establishment of cause-effect in genetic variants requires rigorous experimental testing for functional validation (40). In addition, the implementation of these methods to better understand B- and T-cell biology will be useful in other clinical scenarios, for example, IEI with variants in STAT3 or other related genes such as the IL6ST receptor. In addition, rigorous functional assays can be informative for treatment monitoring, such as JAK inhibitors, in patients with IEI, malignancies, and autoimmune diseases, and *in vitro* B-cell assays can be applied to evaluate primary and secondary antibody deficiencies. Finally, as a limitation of our current study, it will be desirable to reproduce this variant p.(Pro58Thr) and to correct in the patient´s cells *ex-vivo* by using the new strategies of gene editing tools.

## Data Availability

The raw data supporting the conclusions of this article will be made available by the authors, without undue reservation.

## References

[1] Tangye SG Al-Herz W Bousfiha A Cunningham-Rundles C Franco JL Holland SM. Human inborn errors of immunity: 2022 update on the classification from the international union of immunological societies expert committee. J Clin Immunol. (2022) 42:1473–507. doi: 10.1007/s10875-022-01289-3 PMC9244088 35748970

[2] Malphettes M Gérard L Carmagnat M Mouillot G Vince N Boutboul D. Late-onset combined immune deficiency: A subset of common variable immunodeficiency with severe T cell defect. Clin Infect Diseases. (2009) 49:1329–38. doi: 10.1086/606059 19807277

[3] Ameratunga R Edwards ESJ Lehnert K Leung E Woon ST Lea E. The rapidly expanding genetic spectrum of common variable immunodeficiency–like disorders. J Allergy Clin Immunology: In Practice. (2023) 11:1646–64. doi: 10.1016/j.jaip.2023.01.048 36796510

[4] Peng XP Caballero-Oteyza A Grimbacher B. Common variable immunodeficiency: more pathways than roads to rome. Annu Rev Pathol Mech Dis. (2024) 46:45. doi: 10.1146/annurev-pathmechdis- 36266261

[5] Hsieh EWY Bolze A Hernandez JD. Inborn errors of immunity illuminate mechanisms of human immunology and pave the road to precision medicine. Immunol Rev. (2024) 322(1):5–14. doi: 10.1111/imr.v322.1 38308392

[6] del-Pino-Molina L Torres Canizales JM Rodríguez-Pena R López-Granados E. Evaluation of B-cell intracellular signaling by monitoring the PI3K-Akt axis in patients with common variable immunodeficiency and activated phosphoinositide 3-kinase delta syndrome. Cytometry B Clin Cytom. (2020) 100 (4):460–6. doi: 10.1002/cyto.b.21956 32961022

[7] del Pino Molina L Torres Canizales JM Pernía O Rodríguez Pena R Ibanez de Caceres I López Granados E. Defective Bcl-2 expression in memory B cells from CVID patients. Clin Exp Immunol. (2020) 203 (3):341–50. doi: 10.1111/cei.13522 32961586 PMC7874840

[8] del-Pino-Molina L Bravo Gallego LY Soto Serrano Y Reche Yebra K Marty Lobo J González Martínez B. Research-based flow cytometry assays for pathogenic assessment in the human B-cell biology of gene variants revealed in the diagnosis of inborn errors of immunity: a Bruton’s tyrosine kinase case-study. Front Immunol. (2023) 14:1–11. doi: 10.3389/fimmu.2023.1095123 PMC10183671 37197664

[9] Kalina T. Reproducibility of flow cytometry through standardization: opportunities and challenges. Cytometry Part A. (2020) 97:137–47. doi: 10.1002/cyto.a.23901 31593368

[10] Lin JX Leonard WJ. The common cytokine receptor γ chain family of cytokines. Cold Spring Harb Perspect Biol (2018) 10 (9):a028449. 29038115 10.1101/cshperspect.a028449 PMC6120701

[11] Noguchi M Huafang Y Rosenblatt HM Filipovich AH Adelstein S Modi WS. Interleukin-2 receptor γ chain mutation results in X-linked severe combined immunodeficiency in humans. Cell. (1993) 73:147–57. doi: 10.1016/0092-8674(93)90167-o 8462096

[12] Stepensky P Keller B Shamriz O von-Spee-Mayer C Friedmann D Shadur B. T+ NK+ IL-2 receptor γ Chain mutation: a challenging diagnosis of atypical severe combined immunodeficiency. J Clin Immunol. (2018) 38:527–36. doi: 10.1007/s10875-018-0514-y 29948574

[13] Ginn SL Smyth C Wong M Bennetts B Rowe PB Alexander IE. A novel splice-site mutation in the common gamma chain (gammac) gene IL2RG results in X-linked severe combined immunodeficiency with an atypical NK+ phenotype. Hum Mutat. (2004) 23:522–3. doi: 10.1002/humu.9235 15108287

[14] Okuno Y Hoshino A Muramatsu H Kawashima N Wang X Yoshida K. Late-onset combined immunodeficiency with a novel IL2RG mutation and probable revertant somatic mosaicism. J Clin Immunol. (2015) 35:610–4. doi: 10.1007/s10875-015-0202-0 26407811

[15] Kawai T Saito M Nishikomori R Yasumi T Izawa K Murakami T. Multiple reversions of an IL2RG mutation restore T cell function in an X-linked severe combined immunodeficiency patient. J Clin Immunol. (2012) 32:690–7. doi: 10.1007/s10875-012-9684-1 22460439

[16] Hsu AP Pittaluga S Martinez B Rump AP Raffeld M Uzel G. IL2RG reversion event in a common lymphoid progenitor leads to delayed diagnosis and milder phenotype. J Clin Immunol. (2015) 35:449–53. doi: 10.1007/s10875-015-0174-0 PMC4504777 26076747

[17] Bravo-García-Morato M Aracil Santos FJ Briones AC Blázquez Moreno A del Pozo Maté Á Domínguez-Soto Á. New human combined immunodeficiency caused by interferon regulatory factor 4 (IRF4) deficiency inherited by uniparental isodisomy. J Allergy Clin Immunol. (2018) 141:1924–1927.e18. doi: 10.1016/j.jaci.2017.12.995 29408330

[18] Tuovinen EA Grönholm J Öhman T Pöysti S Toivonen R Kreutzman A. Novel hemizygous IL2RG p.(Pro58Ser) mutation impairs IL-2 receptor complex expression on lymphocytes causing X-linked combined immunodeficiency. J Clin Immunol. (2020) 40:503–14. doi: 10.1007/s10875-020-00745-2 PMC7142052 32072341

[19] Kalina T Flores-Montero J van der Velden VHJ Martin-Ayuso M Böttcher S Ritgen M. EuroFlow standardization of flow cytometer instrument settings and immunophenotyping protocols. Leukemia. (2012) 26:1986–2010. doi: 10.1038/leu.2012.122 22948490 PMC3437409

[20] Blanco E Pérez-Andrés M Arriba-Méndez S Contreras-Sanfeliciano T Criado I Pelak O. Age-associated distribution of normal B-cell and plasma cell subsets in peripheral blood. J Allergy Clin Immunol. (2018) 141:2208–2219.e16. doi: 10.1016/j.jaci.2018.02.017 29505809

[21] Le Gallou S Caron G Delaloy C Rossille D Tarte K Fest T. IL-2 requirement for human plasma cell generation: coupling differentiation and proliferation by enhancing MAPK–ERK signaling. J Immunol. (2012) 189:161–73. doi: 10.4049/jimmunol.1200301 22634617

[22] Bryceson YT Pende D Maul-Pavicic A Gilmour KC Ufheil H Vraetz T. A prospective evaluation of degranulation assays in the rapid diagnosis of familial hemophagocytic syndromes. Blood. (2012) 119 (12):2754–63. doi: 10.1182/blood-2011-08-374199 22294731

[23] del Pino Molina L Reche Yebra K Soto Serrano Y Clemente Bernal Á Avendaño-Monje CL Ocejo-Vinyals JG. Technical challenges of intracellular flow cytometry-based assays as a functional complement to diagnosis of signaling defects of inborn errors of immunity: PI3K pathway as a case of study. Front Immunol. (2024) 15. doi: 10.3389/fimmu.2024.1476218 PMC11604744 39620215

[24] Kuijpers TW Baars PA Aan De Kerk DJ Jansen MH Derks IAM Bredius RGM. A novel mutation in CD132 causes X-CID with defective T-cell activation and impaired humoral reactivity. J Allergy Clin Immunol. (2011) 128 (6):1360–3.e4. doi: 10.1016/j.jaci.2011.07.001 21831415

[25] Hou Y Peter Gratz H Ureña-Bailén G Gratz PG Schilbach-Stückle K Renno T. Somatic reversion of a novel IL2RG mutation resulting in atypical X-linked combined immunodeficiency. Genes (Basel). (2021) 13:35. doi: 10.3390/genes 35052377 PMC8774591

[26] Syeda MZ Hong T Huang C Huang W Mu Q. B cell memory: from generation to reactivation: a multipronged defense wall against pathogens. Cell Death Discovery. (2024) 10 (1):117. doi: 10.1038/s41420-024-01889-5 38453885 PMC10920759

[27] Inoue T Kurosaki T. Memory B cells. Nat Rev Immunol. (2024) 24:5–17. doi: 10.1038/s41577-023-00897-3 37400644

[28] Marsman C Verhoeven D Koers J Rispens T ten Brinke A van Ham SM. Optimized protocols for *in-vitro* T-cell-dependent and T-cell-independent activation for B-cell differentiation studies using limited cells. Front Immunol. (2022) 13:815449. doi: 10.3389/fimmu.2022.815449 35844625 PMC9278277

[29] Hipp N Symington H Pastoret C Caron G Monvoisin C Tarte K. IL-2 imprints human naive B cell fate towards plasma cell through ERK/ELK1-mediated BACH2 repression. Nat Commun. (2017) 8 (1):1443. doi: 10.1038/s41467-017-01475-7 29129929 PMC5682283

[30] Chakma CR Good-Jacobson KL. Requirements of IL-4 during the generation of B cell memory. J Immunol. (2023) 210:1853–60. doi: 10.4049/jimmunol.2200922 37276051

[31] Linterman MA Beaton L Yu D Ramiscal RR Srivastava M Hogan JJ. IL-21 acts directly on B cells to regulate Bcl-6 expression and germinal center responses. J Exp Med. (2010) 207:353–63. doi: 10.1084/jem.20091738 PMC2822609 20142429

[32] Zotos D Coquet JM Zhang Y Light A D’Costa K Kallies A. IL-21 regulates germinal center B cell differentiation and proliferation through a B cell-intrinsic mechanism. J Exp Med. (2010) 207:365–78. doi: 10.1084/jem.20091777 PMC2822601 20142430

[33] Nutt SL Hodgkin PD Tarlinton DM Corcoran LM. The generation of antibody-secreting plasma cells. Nat Rev Immunol. (2015) 15:160–71. doi: 10.1038/nri3795 25698678

[34] Fuchs S Rensing-Ehl A Erlacher M Vraetz T Hartjes L Janda A. Patients with T+/low NK+ IL-2 receptor γ chain deficiency have differentially-impaired cytokine signaling resulting in severe combined immunodeficiency. Eur J Immunol. (2014) 44:3129–40. doi: 10.1002/eji.201444689 25042067

[35] Yu X Almeida JR Darko S van der Burg M Deravin SS Malech H. Human syndromes of immunodeficiency and dysregulation are characterized by distinct defects in T-cell receptor repertoire development. J Allergy Clin Immunol. (2014) 133:1109–1115.e14. doi: 10.1016/j.jaci.2013.11.018 24406074 PMC3972286

[36] Kuijpers TW van Leeuwen EMM Barendregt BH Klarenbeek P de Kerk DJ Baars PA. A reversion of an IL2RG mutation in combined immunodeficiency providing competitive advantage to the majority of CD8+ T cells. Haematologica. (2013) 98:1030–8. doi: 10.3324/haematol.2012.077511 PMC3696605 23403317

[37] Tuovinen EA Pöysti S Hamdan F Le KM Keskitalo S Turunen T. Characterization of expanded gamma delta T cells from atypical X-SCID patient reveals preserved function and IL2RG-mediated signaling. J Clin Immunol. (2023) 43:358–70. doi: 10.1007/s10875-022-01375-6 PMC9892142 36260239

[38] Tanita K Hoshino A Imadome KI Kamiya T Inoue K Okano T. Epstein-Barr virus-associated γδ T-cell lymphoproliferative disorder associated with hypomorphic IL2RG mutation. Front Pediatr. (2019) 7. doi: 10.3389/fped.2019.00015 PMC6369201 30778380

[39] Wada F Kondo T Nakamura M Uno S Fujimoto M Miyamoto T. EBV-associated lymphoproliferative disorder in a patient with X-linked severe combined immunodeficiency with multiple reversions of an IL2RG mutation in T cells. EJHaem. (2020) 1:581–4. doi: 10.1002/jha2.v1.2 PMC9175913 35845012

[40] Akalu YT Bogunovic D. Inborn errors of immunity: an expanding universe of disease and genetic architecture. Nat Rev Genet. (2024) 25:184–95. doi: 10.1038/s41576-023-00656-z PMC13489759 37863939

[41] Shearer WT Rosenblatt HM Gelman RS Oyomopito R Plaeger S Richard Stiehm E. Basic and clinical immunology Lymphocyte subsets in healthy children from birth through 18 years of age: The Pediatric AIDS Clinical Trials Group P1009 study. J Allergy Clin Immunol. (2003) 112 (5):973–80. Available online at: www.mosby.com/jaci. 10.1016/j.jaci.2003.07.003 14610491

