# Supplementary material for: Functional insights of an uncommon hypomorphic variant in IL2RG as a monogenic cause of CVID-like disease with antibody deficiency and T CD4 lymphopenia
Source: Front Immunol. 2025 Mar 18;16:1544863. doi: 10.3389/fimmu.2025.1544863 (PMC11958980; doi:10.3389/fimmu.2025.1544863)

Supplementary Figure 1:

CD132 surface expression analyzed in monocytes and granulocytes, selected by forward/scatter properties and CD45^{+} expression (a). The patient presents reduced CD132 levels in comparison to healthy donors as shown in histograms with the median fluorescence intensity (MFI) of CD132 of a representative healthy donor, the patient and the correspondent isotypes controls (b) and box and whiskers (c).

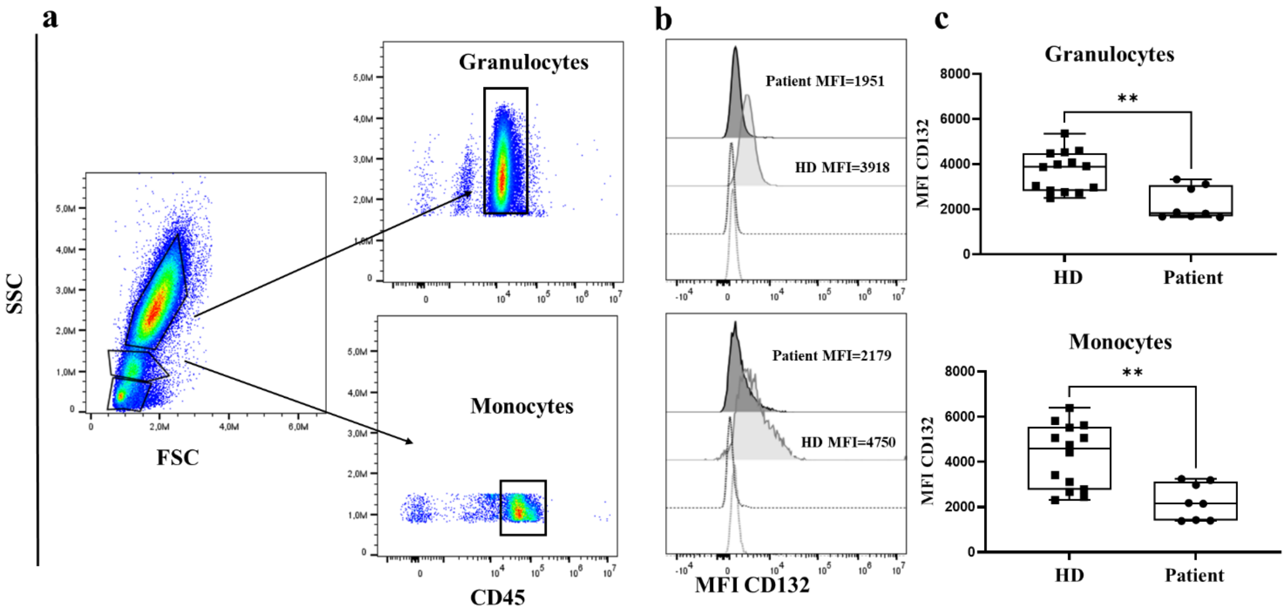

Supplement: Supplementary file 1 [file DataSheet1.pdf]
