# Supplementary material for: Functional insights of an uncommon hypomorphic variant in IL2RG as a monogenic cause of CVID-like disease with antibody deficiency and T CD4 lymphopenia
Source: Front Immunol. 2025 Mar 18;16:1544863. doi: 10.3389/fimmu.2025.1544863 (PMC11958980; doi:10.3389/fimmu.2025.1544863)
Supplement: Supplementary file 2 [file DataSheet2.pdf]

Supplementary Table S1:

Primer name	Sequence (5´ to 3´)
IL2RG exon 1 Fw	CCCGTGTCACACAGCACATA
IL2RG exon 1 Rv	CCCTTCCCACTCCACTTTTCA
IL2RG exon 2 Fw	GCCTCCTCCTTCTGACCAT
IL2RG exon 2 Rv	GAGTTCTTGTACCTAGAGGAGAAA
IL2RG exon 3 Fw	GGTGGGTTGGATCAGAGACC
IL2RG exon 3 Rv	CCTCCTCTTTTCTGCCCATGT
IL2RG exon 4 Fw	GAGACCAGGGATACTGTGGG
IL2RG exon 4 Rv	AGGTCCTTCTATCTGTCTGGT
IL2RG exon 5 Fw	GAGCAGTGTGGCTTGAGTAGT
IL2RG exon 5 Rv	GACAGTGTGGAGAGATGGGG
IL2RG exon 6 Fw	GCAAGGTGAAGATGGCTTTGG
IL2RG exon 6 Rv	CCTGTGGCTTCCTTCCATCA
IL2RG exon 7 Fw	CAGAGGAGGGTTTTGCAGGG
IL2RG exon 7 Rv	TTTGGTCGGCCACATCCTG
IL2RG exon 8 Fw	GAGGCCCTGCACTAACTGTC
IL2RG exon 8 Rv	ACGCAGGTGGGTTGAATGAA

Fw: Forward; Rv: Reverse.
